# Genomic Insights Into Clinical Shiga Toxin-Producing *Escherichia coli* Strains: A 15-Year Period Survey in Jönköping, Sweden

**DOI:** 10.3389/fmicb.2021.627861

**Published:** 2021-02-05

**Authors:** Xiangning Bai, Ji Zhang, Ying Hua, Cecilia Jernberg, Yanwen Xiong, Nigel French, Sture Löfgren, Ingela Hedenström, Anoop Ambikan, Sara Mernelius, Andreas Matussek

**Affiliations:** ^1^Division of Infectious Diseases, Department of Medicine Huddinge, Karolinska Institutet, Huddinge, Sweden; ^2^State Key Laboratory of Infectious Disease Prevention and Control, National Institute for Communicable Disease Control and Prevention, Chinese Center for Disease Control and Prevention, Beijing, China; ^3^^*m*^EpiLab, School of Veterinary Science, Massey University, Palmerston North, New Zealand; ^4^Division of Clinical Microbiology, Department of Laboratory Medicine, Karolinska Institutet, Huddinge, Sweden; ^5^Department of Microbiology, School of Public Health, Southern Medical University, Guangzhou, China; ^6^The Public Health Agency of Sweden, Solna, Sweden; ^7^New Zealand Food Safety Science and Research Centre, School of Veterinary Science, Massey University, Palmerston North, New Zealand; ^8^Laboratory Medicine, Jönköping Region County, Department of Clinical and Experimental Medicine, Linköping University, Jönköping, Sweden; ^9^Division of Laboratory Medicine, Oslo University Hospital, Oslo, Norway; ^10^Division of Laboratory Medicine, Institute of Clinical Medicine, University of Oslo, Oslo, Norway

**Keywords:** Shiga toxin-producing *Escherichia coli*, whole genome sequencing, comparative genomics, bloody diarrhea, clinical outcomes, duration of bacterial shedding

## Abstract

Shiga toxin-producing *Escherichia coli* (STEC) are important foodborne pathogens that can cause human infections ranging from asymptomatic carriage to bloody diarrhea (BD) and fatal hemolytic uremic syndrome (HUS). However, the molecular mechanism of STEC pathogenesis is not entirely known. Here, we demonstrated a large scale of molecular epidemiology and in-depth genomic study of clinical STEC isolates utilizing clinical and epidemiological data collected in Region Jönköping County, Sweden, over a 15-year period. Out of 184 STEC isolates recovered from distinct patients, 55 were from patients with BD, and 129 were from individuals with non-bloody stools (NBS). Five individuals developed HUS. Adults were more associated with BD. Serotypes O157:H7, O26:H11, O103:H2, O121:H19, and O104:H4 were more often associated with BD. The presence of Shiga toxin-encoding gene subtypes *stx*_2a_, *stx*_2a_ + *stx*_2c_, and *stx*_1a_ + *stx*_2c_ was associated with BD, while *stx*_1__a_ was associated with milder disease. Multiplex virulence and accessory genes were correlated with BD; these genes encode toxins, adhesion, autotransporters, invasion, and secretion system. A number of antimicrobial resistance (AMR) genes, such as aminoglycoside, aminocoumarin, macrolide, and fluoroquinolone resistance genes, were prevalent among clinical STEC isolates. Whole-genome phylogeny revealed that O157 and non-O157 STEC isolates evolved from distinct lineages with a few exceptions. Isolates from BD showed more tendency to cluster closely. In conclusion, this study unravels molecular trait of clinical STEC strains and identifies genetic factors associated with severe clinical outcomes, which could contribute to management of STEC infections and disease progression if confirmed by further functional validation.

## Introduction

Shiga toxin-producing *Escherichia coli* (STEC) belong to a genetically and phenotypically diverse group of *E. coli* strains characterized by the production of one or more Shiga toxins (Stx) (Bryan et al., 2015). STEC may cause asymptomatic infection, bloody diarrhea (BD), and life-threatening hemolytic uremic syndrome (HUS) (Bruyand et al., 2018). The most predominant STEC serotype is O157:H7, which has been strongly associated with severe clinical symptoms (Preussel et al., 2013). However, non-O157 STEC strains are increasingly recognized as the main cause of sporadic cases or outbreaks worldwide, especially since 2011 when a STEC O104:H4 outbreak occurred in Germany and spread rapidly in European countries (Bielaszewska et al., 2011). A recent systematic review showed that non-O157 STEC are more common causes of acute diarrhea than the better-known O157 strains (Valilis et al., 2018). The most predominant non-O157 serogroups causing human infections are O26, O45, O103, O111, O121, and O145 in the United States, defined as the “big six” (Gould et al., 2013). These might differ from those of other countries and vary year to year. For example, O80 serogroup, along with O26, has emerged to become a predominant serogroup in 2015 among HUS patients reported in France (Ingelbeen et al., 2018). The latest European annual STEC surveillance report showed that 8,658 confirmed STEC cases were registered in 30 EU/EEA countries in 2018, among which Germany had the highest confirmed STEC cases (2,226), followed by Ireland (966) and Sweden (892) (European Centre for Disease Prevention and Control, 2020). It is noteworthy that a previous study estimated that the true annual number of STEC cases in Germany is 28,347, with a median of 4,969 cases due to O157 STEC and 22,019 cases due to non-O157 STEC, which is much higher than the number registered in European surveillance system (Kuehne et al., 2016). This is probably true for many other European countries as well.

Stx is the key virulence factor of STEC and comprises two types: Stx1 and Stx2 (corresponding genes are referred to as *stx*_1_ and *stx*_2_) (Bergan et al., 2012). The Stx1/Stx2 can further be classified into subtypes, among which Stx2a, Stx2c, and Stx2d were significantly associated with development of HUS, whereas other subtypes were linked to mild symptoms (Scheutz, 2014). Recently, the discovery of the new subtype Stx2k in diarrheal patients indicated the pathogenic potential of uncommon and emerging Stx subtypes (Yang et al., 2020). Besides Stx, other virulence factors also play a role in STEC pathogenicity. For example, intimin encoded by *eae* gene residing on the locus of enterocyte effacement (LEE) pathogenicity island plays a critical role in intestinal colonization. The LEE island encodes a type III secretion system (TTSS), which is responsible for the attaching and effacing (A/E) lesions on intestinal epithelia (Stevens and Frankel, 2014). The presence of both *stx*_2_ and *eae* is associated with a greater probability of triggering severe clinical symptoms (Werber et al., 2003). However, strains carrying *stx* and *eae* do not always cause a severe disease, suggesting the potential role of other virulence factors in the disease progression, which warrants further exploration.

The natural reservoir of STEC is the gastrointestinal tract of ruminant animals, particularly cattle. Human infection is commonly acquired through ingestion of contaminated beef, water, and vegetables or through contact with animals (Mughini-Gras et al., 2018). As the dose of infection is low, person-to-person transmission can also occur. STEC may be shed from the bowel of infected individuals after the resolution of symptoms. Previous studies have shown that patients tested *stx* positive for up to 256 days (Vonberg et al., 2013; Matussek et al., 2016). Long-term STEC carriers represent a chronic risk of person-to-person transmission; thus, their social and working life is legally restricted by the health authorities, posing a high psychological and socioeconomic burden. In Sweden, there is a legal requirement for at least one *stx*_2_-negative stool sample before children are allowed to return to kindergarten (Nolskog et al., 2017). In the United Kingdom, children below 5 years must not return to school until they have two negative stool cultures 24 h apart, while children older than 5 years must have 48 h of negative stool prior to returning to school (Walsh and Johnson, 2018). The possibility of finding molecular predictors for prolonged duration of shedding is of great importance to optimize control measures to limit the spread from person to person.

Previous studies have contributed to a deeper insight in the STEC infections in different populations in Europe, as well as a general understanding of pathogenic STEC strains associated with human infections (Buvens et al., 2012; Messens et al., 2015; Fierz et al., 2017; De Rauw et al., 2018; Nuesch-Inderbinen et al., 2018). We have previously reported STEC infection in adults over 10 years of age in Jönköping, Sweden, and we depicted the main virulence factors associated with clinical outcomes (Bai et al., 2018b). An earlier microarray analysis on targeted virulence factors of STEC isolates from children below 10 years of age showed that multiplex virulence genes were associated with BD compared with cases with non-BD, and several genes were associated with prolonged duration of bacterial shedding (Matussek et al., 2017). An in-depth analysis is essential to unveil the complete picture of how genetic factors correlate with clinical outcomes. In the current study, we investigated the molecular epidemiology of STEC isolates from infected individuals including diarrheal patients and contacts identified through contact tracing from April 2003 through 2017, in Region Jönköping County, Sweden. Whole-genome sequencing was done on all isolates to characterize the molecular features. Bacterial genomic data were analyzed in combination with the clinical and epidemiological data to explore the genetic factors that might be associated with severe clinical outcomes, and the phylogenetic relatedness of all isolates was assessed.

## Materials and Methods

### Collection of Shiga Toxin-Producing *Escherichia coli* Isolates and Metadata

In total, 195 STEC isolates were collected from diarrheal patients and individuals identified after tracing contacts of index cases from April 2003 through 2017 in Region Jönköping County, Sweden. Clinical data from STEC patients are collected through routine praxis used for the STEC surveillance performed in Region Jönköping County, Sweden. Formal consent is not required, as sample and data collection are part of the routine microbiological and contact tracing work in Region Jönköping County, Sweden.

### Associations Between Age, Country of Infection, Duration of *stx* Shedding, and Bloody Diarrhea

The relationships between age and country of infection and the binary outcome variables BD (yes or no) and duration of *stx* shedding (>24 or ≤24 days) were assessed by considering these variables as covariates in mixed-effects logistic regression models, with outbreak group (OG) as a random effect to allow for the lack of statistical independence between cases belonging to the same outbreak. Models were fitted using the package lme4 in R (Bates et al., 2015).

### Whole-Genome Sequencing and Assembly

For the 167 STEC isolates collected from April 2003 through 2014, bacterial genomic DNA was extracted using the EZ1 DNA Tissue Kit on EZ1 instrument (Qiagen, Hilden, Germany) according to the manufacturer’s protocol. DNA library preparation was done using Nextera chemistry (Illumina). The library was then pair-end (2 × 150 bp) sequenced using the Illumina HiSeq X platform (paired-end reads; read length 150 bp) at SciLifeLab (Stockholm, Sweden). For the 28 STEC isolates collected from 2015 through 2017, DNA was extracted using the MagDEA Dx SV reagent kit on magLEAD instrument (Precision System Science, Chiba, Japan). The DNA library preparation and purification were done using the AB Library Builder system and AMPure beads (Thermo Fisher Scientific, Waltham, MA, United States). The library was then sequenced using the Ion Torrent S5 XL platform (single-end reads; length 400 bp) at The Public Health Agency of Sweden as previously described (Lagerqvist et al., 2020). The quality of the raw reads was assessed with FastQC (version 0.11.8)^1^. Trimmomatic (version: 0.38) was used to trim the adapter sequences and low-quality bases (quality scores 10) from the beginning and end of the sequencing reads. Sequencing reads that were shorter than 30 bp were eliminated from further analyses (Bolger et al., 2014). The Illumina sequencing reads were *de novo* assembled with SKESA (version 2.3.0) with the adapter sequence mechanism disabled (Souvorov et al., 2018). The Ion Torrent sequencing reads were *de novo* assembled with SPAdes (version 3.12.0) in “careful mode” (Bankevich et al., 2012). The draft genome sequences were annotated with Prokka (version 1.11) (Seemann, 2014) using the built-in *Escherichia*-specific BLAST database. Eleven isolates failed in sequencing or yielded low-quality reads, which were excluded in subsequent analyses; thus, 184 STEC isolates were analyzed in this study.

### Determination of *stx* Subtypes and Serotypes

The *stx* subtypes of STEC isolates were determined by ABRicate version 0.8.10^2^ using default parameters. Briefly, an in-house *stx* subtyping database was created with ABRicate by integrating representative nucleotide sequences of all identified *stx*_1_ and *stx*_2_ subtypes, which included *stx*_1_ and *stx*_2_ subtypes previously reported by Scheutz et al. (2012); two recently identified *stx*_2_ subtypes, *stx*_2__h_ (Bai et al., 2018a) and *stx*_2__m_ (Yang et al., 2020); and one provisional subtype, *stx*_2__i_ (Lacher et al., 2016). The assemblies were then used to search against the *stx* subtyping database. Serotype was determined by comparing assemblies with the SerotypeFinder database using BLAST + v2.2.30 (Camacho et al., 2009).

### Characterization of Virulence Factors and Antimicrobial Resistance Genes

The VFDB database^3^ was used for determination of virulence factors, and the CARD database^4^ was used for AMR factors. Gene presence/absence was determined using ABRicate version 0.8.10 with the following parameters: coverage ≥ 60% and identity ≥ 80%. The statistical association between virulence/AMR genes and isolate classifications was assessed with Fisher’s exact test using Statistica12 (StatSoft, Inc. Tulsa, OK, United States) for three classification models (age groups, clinical symptoms, and duration of *stx* shedding). Factors with corrected *p*-value (Benjamini–Hochberg) of association below 0.05 were considered statistically significant. For the clinical relevance of specific *stx* subtypes, Fisher’s exact test was done separately in different groups, with a *p*-value < 0.05 regarded as statistically significant.

### Pan Genome-Wide Association Study

The pan genomes of 184 STEC isolates were calculated from the harmonized genome annotations produced by Prokka, using Roary^5^. The accessory genes were associated with clinical variables (age, clinical symptoms, and duration of *stx* shedding) using Scoary v1.6.16 (run with 1,000 permutation replicates) (Brynildsrud et al., 2016). Patterns of genes were reported as statistically significantly associated with a variable if they attained a Benjamini–Hochberg-corrected *p*-value of below 0.05. Given isolates from the same OG, which had an epidemiological link with each other, may introduce confounding, Scoary was further performed on a subset of isolates, including only the index case in each OG. In addition, multiple correspondence analysis (MCA) of pan genomes was performed using the gene presence/absence table generated from Roary. The R function MCA from R package FactoMineR was used for the analysis (Lê et al., 2008). The gene presence/absence table of all isolates was represented as categorical data. The total dimension of the data was reduced to 20 dimensions, and the first two dimensions that captured the highest variation were used to visualize the clustering. Default values were used for the rest of the parameters.

### Whole-Genome Phylogenetic Analysis

The phylogenetic relationships of all 184 STEC isolates, together with six reference *Escherichia coli* genomes of predominant STEC serotypes (Supplementary Figure S1), were assessed by whole-genome multilocus sequence typing (wgMLST) and whole-genome phylogeny analysis. To define wgMLST allelic profiles, we used Fast-GeP^6^ (Zhang et al., 2018) with default settings, using the complete genome sequence of strain EDL933 (Acc. CP008957.1) as a reference. The whole-genome polymorphic sites-based phylogeny was inferred from the concatenated sequences of the coding sequences (CDSs) shared by all the whole-genome sequences. All the regions with elevated densities of base substitutions were eliminated, and a final maximum likelihood tree was generated by Gubbins (version 2.4.1) (Croucher et al., 2015) with default settings. The phylogenetic tree was annotated with relevant metadata using iTOL^7^.

### Data Availability

The draft genome assemblies were submitted to GenBank under the BioProject PRJNA630106. Four strains included in this study have been previously described (Bai et al., 2019).

## Results

### Shiga Toxin-Producing *Escherichia coli* Cases

Among 184 STEC isolates, 55 were from patients with BD, out of which four developed HUS. There were 129 isolates from individuals with non-bloody stools (NBS) including non-BD patients and asymptomatic carriers identified through contact contracting around an index case, out of which one developed HUS. Duration of *stx* shedding was available in 130 STEC-infected individuals. The median duration of *stx* shedding was 24 days (0–294 days), which was used to separate short (≤24 days) and long (> 24 days) duration of shedding. Sixty-two individuals exhibited a long duration of shedding, and 68 showed a short duration of shedding. Among the 184 STEC-infected individuals, 104 were children (<10 years old) and 80 were adults (≥10 years old). Contact tracing information was available for 73 out of 184 infected individuals, of which 32 were index cases. The index case and contacts, if any, were grouped into one OG; 48 OGs were assigned in total, of which 18 comprised more than one individual. After country of origin and OG were adjusted, adults were more associated with BD than children (odds ratio 4.8, 95% CI 1.2–18.9, *p* = 0.026), while children showed longer duration of *stx* shedding, which was not significant after adjusting for age, country of infection, and OG (*p* = 0.059) (Table 1). The travel history was available for all individuals, among which 123 were domestically infected while 61 were travel-associated. The possible source of infection was primarily from consumption of products from food-producing animals, such as milk, cheese, and sausage. The metadata of STEC isolates are shown in Supplementary Table S1.

**TABLE 1:** Characteristics of STEC-infected individuals included in this study.

	Clinical symptoms				Duration of *stx* shedding			
Group	BD	NBS	OR*	*p*-value*	Long	Short	OR*	*p*-value*
	(*n* = 55)	(*n* = 129)	(95% CI)		(*n* = 62)	(*n* = 68)	(95% CI)	
**Age group**								
Child (<10 years)	20 (36.37)	84 (65.12)	1.0 (ref)	0.026**	48 (77.42)	41 (60.30)	1.0 (ref)	0.059
Adult (≥10 days)	35 (63.67)	45 (34.88)	4.8 (1.2–18.9)		14 (22.58)	27 (39.70)	0.4 (0.2–1.0)	
**Country of infection**								
Sweden	42 (76.36)	81 (62.79)	1.0 (ref)	0.38	38 (61.29)	45 (66.18)	1.0 (ref)	0.69
Abroad	13 (23.64)	48 (37.21)	1.6 (0.6–4.5)		24 (38.71)	23 (33.82)	0.9 (0.4–1.8)	
Duration of *stx* shedding***								
Long (>24 days)	17 (47.22)	45 (47.87)	1.0 (ref)	0.83	–	–	–	–
Short (≤24 days)	19 (52.78)	49 (52.13)	1.1 (0.4–3.0)		–	–	–	–

BD, bloody diarrhea; NBS, non-bloody stools. *Odds ratios (ORs) and *p*-values after adjusting for confounding by including both age group and country of infection as fixed effects and outbreak number as a random effect in mixed-effects logistic regression models. **Statistically significant difference. ***There were 54 missing values for this variable (19 for BD and 35 for NBS).

### Shiga Toxin-Producing *Escherichia coli* Serotypes and *stx* Subtypes in Correlation to Clinical Symptoms

**FIGURE 1:**
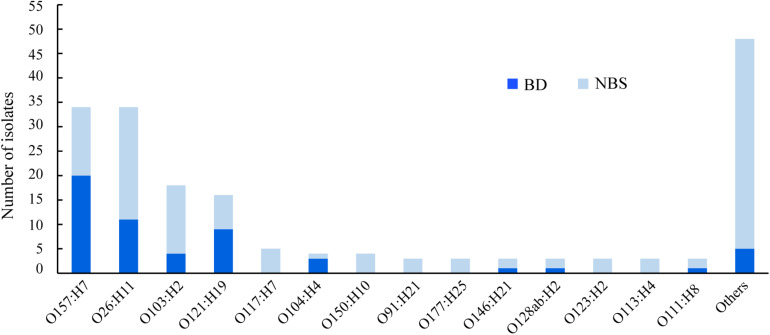
Serotypes of clinical Shiga toxin-producing *Escherichia coli* (STEC) isolates in correlation with clinical symptoms. BD, patients with bloody diarrhea; NBS, individuals with non-bloody stools.

In total, 51 different O:H serotypes were assigned among 184 STEC isolates. The most predominant serotypes were O157:H7 and O26:H11, comprising 34 isolates each, followed by O103:H2 and O121:H19, comprising 18 and 16 isolates, respectively (Figure 1 and Supplementary Table S1). The majority of isolates of serotypes O157:H7, O121:H19, and O104:H4 were correlated to BD (Figure 1). Two O104:H4 isolates, one O121:H19, one O98:H21, and one O157:H7 isolate were isolated from HUS patients.

Seventeen different *stx* subtypes/combinations were identified, with *stx*_1a_ (71 isolates), *stx*_2a_ (27), *stx*_2a_ + *stx*_2c_ (20), and *stx*_2c_ (11) being most predominant, followed by *stx*_1c_ (9 isolates), *stx*_1a_ + *stx*_2c_ (8), *stx*_1c_ + *stx*_2b_ (8), and *stx*_2b_ (8). The presence of *stx*_2a_, *stx*_2a_ + *stx*_2c_, and *stx*_1a_ + *stx*_2c_ was statistically associated with BD, while *stx*_1a_ was associated with NBS(*p* < 0.05) (Table 2). The presence of *stx*_1a_ and *stx*_2c_ was statistically associated with children group, while *stx*_1a_ + *stx*_2a_ was associated with the adults group (Table 2). No association was observed between *stx* types/subtypes and duration of *stx* shedding. Three out five isolates from HUS patients carried *stx*_2a_, one carried *stx*_2a_ + *stx*_2c_, and one possessed *stx*_1a_. A correlation was observed between serotypes and *stx* subtypes. All 18 isolates of O103:H2 serotype carried the *stx*_1a_ subtype; all 16 isolates of O121:H19 serotype carried the *stx*_2a_ subtype, with one isolate carrying both *stx*_2a_ and *stx*_1a_; 31 out of 34 O26:H11 isolates carried the *stx*_1a_ subtype.

**TABLE 2:** Prevalence of *stx* types/subtypes in correlation with clinical symptoms^*a*^, age group^*b*^, and duration of *stx* shedding^*c*^.

*Stx* type/subtype	Clinical symptoms		*p*-value	Age groups			Duration of *stx* shedding		*p-*value
	BD (*n* = 55)	NBS (*n* = 129)		Adult (*n* = 80)	Child (*n* = 104)	*p*-value	L (*n* = 62)	S (*n* = 68)	
***stx* type**									
*stx*_1_ only	14 (25.5)	67 (51.9)	0.001*	25 (31.3)	56 (53.8)	0.002*	33 (53.2)	29 (42.6)	0.228
*stx*_2_ only	29 (52.7)	46 (35.7)	0.031*	38 (47.5)	37 (35.6)	0.103	20 (32.2)	30 (44.1)	0.165
*stx*_1_ + *stx*_2_	12 (21.8)	16 (12.4)	0.104	17 (21.3)	11 (10.6)	0.046*	9 (14.5)	9 (13.2)	0.833
***stx* subtype**									
*stx*_1__a_	14 (25.5)	57 (44.2)	0.017*	21 (26.3)	50 (48.1)	0.003*	31 (50.0)	27 (39.7)	0.238
*stx*_2__a_	14 (25.5)	13 (10.1)	0.007*	14 (17.5)	13 (12.5)	0.342	9 (14.5)	7 (10.3)	0.464
*stx*_2__a_ + *stx*_2__c_	13 (23.6)	7 (5.43)	0.00028*	12 (15.0)	8 (7.69)	0.114	5 (8.06)	8 (11.8)	0.482
*stx*_2__c_	1 (1.82)	10 (7.75)	0.178	1 (1.25)	10 (9.61)	0.025*	3 (4.84)	8 (11.8)	0.156
*stx*_1__c_	0	9 (6.98)	0.59	4 (5.00)	5 (4.81)	0.952	2 (3.22)	1 (1.47)	0.605
*stx*_1__c_ + *stx*_2__b_	1 (1.82)	7 (5.43)	0.439	4 (5.00)	4 (3.85)	0.704	3 (4.84)	1 (1.47)	0.347
*stx*_2__b_	1 (1.82)	7 (5.43)	0.439	4 (5.00)	4 (3.85)	0.704	2 (3.23)	2 (2.94)	1
*stx*_1__a_ + *stx*_2__c_	6 (10.9)	2 (1.55)	0.01*	4 (5.00)	4 (3.85)	0.704	2 (3.23)	4 (5.88)	0.628
*stx*_1__a_ + *stx*_2__a_	5 (9.09)	2 (1.55)	0.026*	6 (7.50)	1 (0.96)	0.044*	2 (3.23)	2 (2.94)	1.0
*stx*_2__d_	0	4 (3.10)	0.319	3 (3.75)	1 (0.96)	0.199	0	3 (4.41)	0.246
*stx*_1__a_ + *stx*_2__b_	0	2 (1.55)	1	2 (2.50)	0	0.105	1 (1.61)	0	1
*stx*_1__a_ + *stx*_2__d_	0	2 (1.55)	1	1 (1.25)	1 (0.96)	0.852	1 (1.61)	1 (1.47)	1
*stx*_2__e_	0	2 (1.55)	1	2 (2.50)	0	0.105	NA	NA	NA
*stx*_2__g_	0	2 (1.55)	1	1 (1.25)	1 (0.96)	0.852	0	2 (2.94)	0.497
*stx*_1__c_ + *stx*_2__d_	0	1 (0.78)	1	0	1 (0.96)	0.379	0	1 (1.47)	1
*stx*_1__d_	0	1 (0.78)	1	0	1 (0.96)	0.379	0	1 (1.47)	1
*stx*_2__b_ + *stx*_2__d_	0	1 (0.78)	1	1 (1.25)	0	0.253	1 (1.61)	0	0.481

NA, data unavailable. ^*a*^Clinical symptoms include bloody diarrhea (BD) and non-bloody stools (NBS). ^*b*^Adult, ≥10 years; child, <10 years. ^*c*^Duration of shedding was available in 130 STEC-infected individuals; L, >24 days; S, ≤24 days. *Statistically significant difference.

### Distribution of Virulence and Antimicrobial Resistance Factors in Clinical Shiga Toxin-Producing *Escherichia coli* Strains

STEC isolates from patients with BD demonstrated a high frequency of carriage for the greatest number of virulence genes. Using Fisher’s exact test corrected for multiple testing with the Benjamini–Hochberg-corrected *p-*value, we found 130 genes that were significantly associated with disease severity, among which 123 genes were positively associated with BD when compared with NBS, while seven genes were inversely associated with BD. These virulence factors can be classified into groups based on their functions: adhesin (*eae*, *etpI*, *lifA*, *paa*, and *toxB*), autotransporters (*ehaA*, *espP*, and *upaC*), toxins (*stx*_1_, *stx*_2_, *astA*, *east1*, and *senB*), invasion (*tia*), iron uptake factors, LEE-encoded TTSS and non-LEE-encoded TTSS effectors, hypothetical proteins, and other factors (Supplementary Table S2). However, no association was found between the virulence factors and prolonged duration of *stx* shedding or age groups.

Seventy AMR genes were identified in 184 STEC isolates, and 34 AMR genes were present in all isolates, which are associated with resistance to 10 classes of antimicrobial drugs, i.e., aminoglycoside, aminocoumarin, macrolide, fluoroquinolone, nucleoside, peptide, penam, tetracycline, fosfomycin, and nitroimidazole. Genes associated with resistance to fluoroquinolone antibiotic, such as *crp*, *hns*, *acrB*, *marA*, *mdtM*, and *emrA* were most commonly detected (Supplementary Table S3). Multidrug resistance gene *emrE* was statistically significantly associated with serotype O157:H7 (*p* < 0.001) (Table 3). *fosA7* only existed in two O5:H9 strains, *sat-1* and *bla*_*TEM–*__150_ genes were only observed in one O35:H10 strain, and *dfrA5* was only found in two O96:H19 strains (Supplementary Table S3).

**TABLE 3:** Prevalence of AMR genes in 184 clinical STEC isolates in correlation with clinical and bacterial variables^a^.

Gene^b^	Duration of *stx* shedding		*p-*value^c^	*p-*value^d^	Serogroups		*p-*value^c^	*p-*value^d^	Age groups		*p-*value^c^	*p-*value^d^
									
	S (*n* = 68)	L (*n* = 62)			Non-O157 (*n* = 150)	O157 (*n* = 34)			Child (*n* = 104)	Adult (*n* = 80)		
*ANT(3″)-IIa*	3 (4.41)	0	0.094	1	3 (2.0)	0	0.406	1	3 (2.88)	0	0.126	1
*APH(3″)-Ib*	6 (8.82)	10 (16.13)	0.205	1	19 (12.67)	5 (14.71)	0.75	1	16 (15.38)	8 (10.0)	0.282	1
*APH(4)-Ia*	1 (1.47)	0	0.338	1	1 (0.67)	0	0.633	1	1 (0.96)	0	0.379	1
*APH(6)-Id*	6 (8.82)	10 (16.13)	0.205	1	19 (12.67)	5 (14.71)	0.75	1	16 (15.38)	8 (10.0)	0.282	1
*bla*_*CTX–M–*__15_	1 (1.47)	2 (3.23)	0.506	1	5 (3.33)	0	0.28	1	1 (0.96)	4 (5.0)	0.095	1
*ampC*	68 (100)	62 (100)	–	–	150 (100)	32 (94.12)	0.033*	0.759	104 (100)	78 (97.5)	0.105	1
*ampC1*	68 (100)	59 (95.16)	0.066	1	144 (96.0)	34 (100)	0.236	1	101 (97.12)	77 (96.25)	0.743	1
*emrE*	38 (55.88)	21 (33.87)	0.012*	0.248	60 (40.0)	28 (82.35)	<0.001*	<0.001*	40 (38.46)	48 (60.0)	0.004*	0.086
*bla*_*TEM–*__1_	4 (5.88)	5 (8.06)	0.624	1	12 (8.0)	1 (2.94)	0.299	1	7 (6.73)	6 (7.5)	0.84	1
*acrE*	68 (100)	59 (95.16)	0.066	1	145 (96.67)	34 (100)	0.28	1	101 (97.12)	78 (97.5)	0.874	1
*acrF*	65 (95.59)	59 (95.16)	0.908	1	144 (96.0)	32 (94.12)	0.627	1	98 (94.23)	78 (97.5)	0.281	1
*acrS*	67 (98.53)	61 (98.39)	0.947	1	148 (98.67)	34 (100)	0.498	1	102 (98.08)	80 (100)	0.212	1
*dfrA7*	1 (1.47)	1 (1.61)	0.947	1	4 (2.67)	0	0.336	1	0 (0.0)	4 (5.0)	0.034*	0.782
*emrK*	67 (98.53)	61 (98.39)	0.947	1	148 (98.67)	34 (100)	0.498	1	102 (98.08)	80 (100)	0.212	1
*emrY*	67 (98.53)	61 (98.39)	0.947	1	148 (98.67)	34 (100)	0.498	1	102 (98.08)	80 (100)	0.212	1
*evgA*	68 (100)	62 (100)	–	–	149 (99.33)	34 (100)	0.633	1	103 (99.04)	80 (100)	0.379	1
*mdtM*	68 (100)	59 (95.16)	0.066	1	144 (96.0)	34 (100)	0.236	1	101 (97.12)	77 (96.25)	0.743	1
*mphB*	66 (97.06)	58 (93.55)	0.341	1	139 (92.67)	34 (100)	0.103	1	100 (96.15)	73 (91.25)	0.164	1
*sul1*	2 (2.94)	3 (4.84)	0.574	1	7 (4.67)	0	0.199	1	3 (2.88)	4 (5.0)	0.457	1
*sul2*	7 (10.29)	10 (16.13)	0.324	1	19 (12.67)	5 (14.71)	0.75	1	15 (14.42)	9 (11.25)	0.526	1
*tet(A)*	5 (7.35)	5 (8.06)	0.879	1	13 (8.67)	1 (2.94)	0.256	1	8 (7.69)	6 (7.5)	0.961	1
*tet(B)*	3 (4.41)	2 (3.23)	0.725	1	4 (2.67)	1 (2.94)	0.929	1	4 (3.85)	1 (1.25)	0.283	1
*ugd*	62 (91.18)	60 (96.77)	0.185	1	138 (92)	34 (100)	0.088	1	95 (91.35)	77 (96.25)	0.182	1

S, short duration of *stx* shedding (≤ 24 days); L, long duration of *stx* shedding (> 24 days). ^*a*^Association was analyzed between the presence of AMR genes and clinical symptoms, duration of *stx* shedding, age groups, and serogroups; the only difference showing statistical significance is shown in this table. ^*b*^34 AMR genes present in all 184 isolates and 13 genes present in less than three isolates were excluded in statistical analysis. ^*c*^The naïve *p*-value. ^*d*^The Benjamini–Hochberg-corrected *p*-value. *Statistically significant difference.

### Genome-Wide Association Study of Genetic Markers for Clinical Significance

**FIGURE 2:**
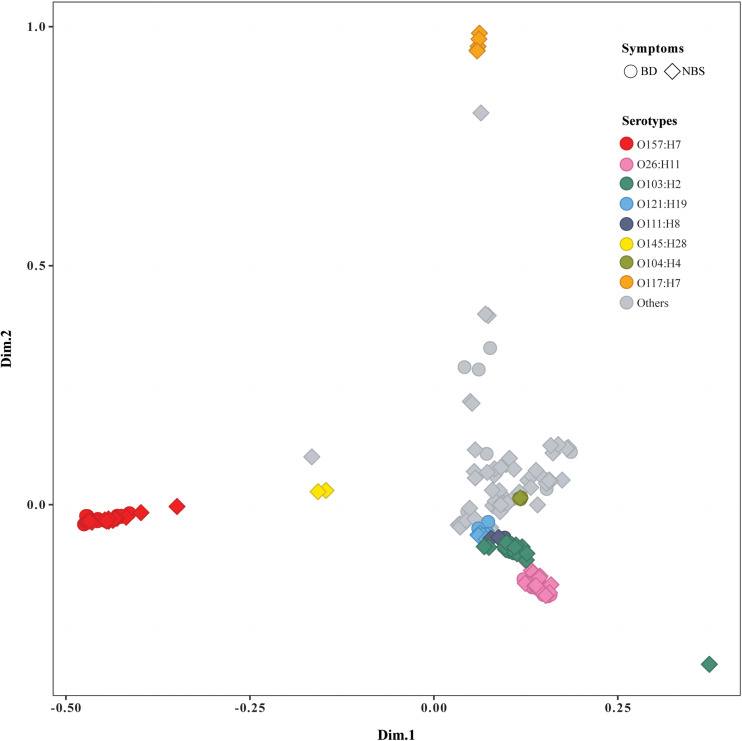
Multiple correspondence analysis plot comparing pan genomes of 184 Shiga toxin-producing *Escherichia coli* (STEC) isolates. BD, patients with bloody diarrhea; NBS, individuals with non-bloody stools. Isolates from BD and NBS are indicated by the ring and square, respectively. The main serotypes were marked in different colors, as indicated.

The pan genome of 184 STEC isolates was composed of 26,474 genes. More than 800 accessory genes were found to be statistically significantly associated with BD, while no gene was statistically significantly associated with age groups or prolonged duration of *stx* shedding. Most of BD-associated genes encoded hypothetical proteins with unknown function (Supplementary Table S4a). Similarly, Scoary on a subset of 163 isolates including only index case from each OG showed that no accessory gene was statistically associated with age or duration of *stx* shedding, and multiple genes were statistically associated with BD compared with NBS (Supplementary Table S4b). MCA of pan genomes showed that all O157:H7 strains clustered together, while O26:H11 strains clustered closely with O103:H2, O121:H19, and other non-O157 serotypes. Five O117:H7 strains clustered separately from others. Isolates from BD and NBS groups scattered throughout different clusters except O117:H7 cluster, which included five strains from NBS (Figure 2). No clear separation was observed between groups of other clinical variables such as duration of *stx* shedding, age, and country of infection (data not shown).

### Phylogenomic Relationships

**FIGURE 3:**
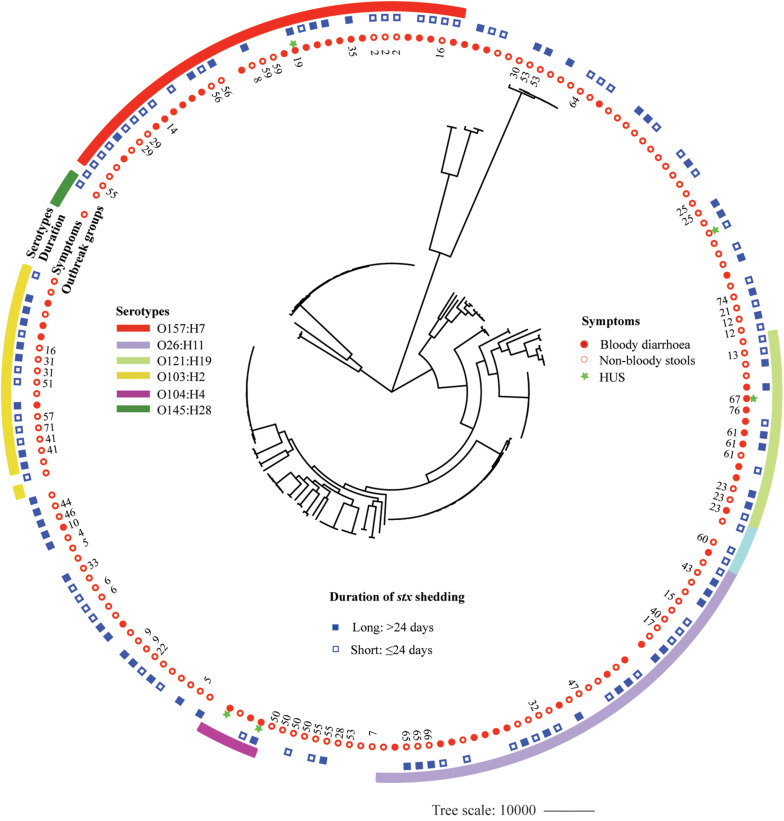
Whole-genome phylogeny of Shiga toxin-producing *Escherichia coli* (STEC) isolates. Circular representation of the Gubbins phylogenetic tree generated from the concatenated sequences of the shared loci found in the wgMLST analysis. Gubbins tree was annotated with relevant metadata using iTOL. From the outer to inner circle, each represents serotypes, duration of bacterial shedding, clinical symptoms, and outbreak groups.

A whole-genome phylogeny was constructed from alignment of concatenated CDSs of the 2,485 shared-loci found in all 184 STEC isolates and six *Escherichia coli* reference genomes representing main STEC serotypes (O157:H7, O145:H28, O111: H-, O26:H11, O104:H4, and O103:H2). The 184 STEC isolates were divided into two clades, O157 and non-O157, except that two O145:H28 isolates and one O8:H19 isolate clustered into O157 clade. Non-O157 clade further formed two subclades, with one comprising most isolates (136) and the other containing 11 isolates. Isolates with the same serotype clustered together. Within the O157:H7 clade, isolates carrying both *stx*_2__a_ and *stx*_2__c_ clustered closely (Supplementary Figure S1). Isolates from BD patients were mostly distributed in six clusters of predominant STEC serotypes, i.e., O157:H7, O26:H11, O121:H19, O103:H2, O111:H8, and O104:H4. However, isolates from patients with long duration of *stx* shedding scattered across different clusters. Out of 48 OGs, 18 included more than one isolate. Isolates from the same OG were grouped together, with four exceptions, i.e., OG55, OG16, OG53, and G5 (Figure 3).

## Discussion

In Sweden, it has been mandatory to report human O157 STEC human infection since 1996 and all serotypes since July 2004. The number has been increasingly reported ever since all serotypes became mandatory to register in 2004 (Nolskog et al., 2017). To our knowledge, this is the first systematic genomic study of clinical STEC isolates since all serotypes have been reported in Sweden. Our study showed that non-O157 serotypes caused 81.5% of STEC infection in Region Jönköping County, Sweden, while O157 serotypes accounted for 18.5% of STEC cases, highlighting the clinical relevance of non-O157 STEC serotypes. We found that O157 and several predominant non-O157 serotypes, such as O26:H11, O103:H2, and O121:H19, were more often associated with severe symptoms such as BD. The incidence of HUS among STEC-infected individuals was 2.7% in this study. Among the five HUS cases, two were caused by serotype O104:H4, and the other three were caused by O157:H7, O121:H19, and O98:H21. This indicates a high pathogenic risk of non-O157 serotypes. It is noteworthy that two out of four O104:H4 isolates were from HUS patients who were part of the German outbreak in 2011 and clustered together with German outbreak strain 2011C-3493.

The presence of *stx*_2__a_ (with or without *stx*_2__c_) is responsible for severe clinical outcomes such as HUS (Orth et al., 2007). It is noteworthy that one O98:H21 isolate from a HUS patient in this study carried only *stx*_1__a_, indicating the potential risk associated with less virulent *stx* subtype strains. Notably, the presence of *stx*_1__a_ and *stx*_2__c_ was associated with children group, while *stx*_1__a_ + *stx*_2__a_ was associated with the adults group. Thus, the association between the adults group and BD observed in this study may be confounded by *stx* subtypes and other factors, for instance, the selection bias in the patients who seek care and were reported with STEC. Adults may be overrepresented in those who have BD, since adults are less likely to seek care for non-BD and other mild symptoms. Besides *stx*, 130 virulence factors were found to be associated with disease severity, among which 123 genes were overrepresented in isolates from patients with BD, while seven genes were related with NBS. Most BD-associated genes encoded TTSS, the best-characterized system disrupting host cell defense by delivering virulence factors into host cells (Pinaud et al., 2018). Intimin gene *eae*; TTSS-related genes *espB*, *tir*, *map*, and *cesF*; and regulation gene *tir*, which are commonly associated with high virulent O157:H7 serotype, were also distributed widely among non-O157 isolates in this study, suggesting a high pathogenicity of some non-O157 isolates. The EspF, which has emerged as a “Swiss army knife” of STEC pathogenesis, can target host mitochondria and the nucleolus, disrupt tight junctions, and induce hemorrhagic enteritis (Holmes et al., 2010). Interestingly, we found that *espF* was more frequent among non-O157 than O157 isolates, which was in accordance with a recent study (Baba et al., 2019). Notably, four isolates harbored heat-stable toxins (ST) encoding genes *sta* and *stb*, the virulence determinants for enterotoxigenic *Escherichia coli* (ETEC), exhibiting STEC/ETEC hybrid pathotype as previously reported (Bai et al., 2019). Further studies are warranted to understand the expression level of these genetic factors and their associations with disease severity.

The use of antibiotics is not recommended in STEC infections, as it may increase the risk of HUS development by inducing Stx production (Bielaszewska et al., 2012). However, AMR is a growing concern due to the widespread of *E. coli* resistant to all antibiotics used in human therapy and the dissemination of AMR genes through mobile genetic elements, which could lead to multiple drug resistance (MDR) (Oporto et al., 2019). In this study, 70 AMR genes were found in 184 clinical STEC isolates, and 25.7% genes were associated with MDR. The antibiotics resistance phenotype remains to be tested to understand the associations of AMR genotypes and phenotypes. For instance, most of the strains in this study carried beta-lactamase family gene *ampC*, which is widely distributed in Enterobacteriaceae. However, the chromosomal *ampC* genes are expressed constitutively at a low level in *Escherichia coli* and confers resistance to cefoxitin only when it is overproduced (Jaurin et al., 1981;Peter-Getzlaff et al., 2011). Fluoroquinolone resistance genes were most frequent (31.4%), which is noteworthy, as fluoroquinolones are commonly used to treat travelers’ diarrhea (Day et al., 2017). The *emrE* gene, which is related to macrolide antibiotic resistance, was overrepresented in isolates from short bacterial shedding and adults, although the difference was not statistically significant after Benjamini–Hochberg correction. A previous study showed that azithromycin, which belongs to ımacrolide antibiotic drug class, might be used safely to treat STEC O104:H4 long-term carriage (Nitschke et al., 2012). However, as the authors pointed out, this finding warrants confirmation for other STEC strains.

The whole-genome phylogeny demonstrated a distinctive evolutionary path between O157 and non-O157 STEC strains with a few exceptions; i.e., three O145:H28 strains clustered closely with O157 strains, which was in agreement with an earlier study showing that O145:H28 demonstrates a common evolutionary lineage with O157:H7 (Cooper et al., 2014). Interestingly, we found that one O8:H19 isolate from a non-BD patient also clustered in O157:H7 clade. These findings were supported by MCA of pan genomes showing that two O145:H28 strains and one O8:H19 strain clustered closely with O157:H7 strains. Further study is needed with more strains to understand if the O8:H19 strains share the same evolutionary path with O157:H7. We found that isolates from BD patients were mostly distributed in the six clusters comprising most predominant and clinical relevant serotypes. Isolates from the same OG clustered closely, indicating that they share a similar genome background. Isolates from long-term carriers scattered throughout different clusters, which to some extent support our findings that no bacterial genetic factor was significantly associated with duration of bacterial shedding. To the best of our knowledge, this is first study investigating the associations between bacterial genetic factors in the genome level and duration of bacterial shedding; our study implies that prolonged bacterial carriage in the gut might be more associated with host-related factors, which would be a valuable future work.

The current study has several limitations. The metadata such as duration of bacterial shedding and contact tracing of some individuals were lacking, which hampers a comprehensive understanding of associations between bacterial genetic factors and clinical traits. This study had selection bias in the patients who seek care and were reported with STEC. Adults are less likely to seek care for non-BD and, thus, may be overrepresented in those who have BD; this might confound the statistical associations between age of patients and bacterial genetic factors such as *stx* subtype. Moreover, statistical associations calculated between genetic factors in all STEC isolates and clinical variables might be skewed by isolates from the same OG, which had a close epidemiological link to each other. The statistical strategy in this study was different from that of our previous report where a naïve *p*-value was applied to find, as many as possible genes, potentially associated with clinical symptoms (Matussek et al., 2017). In this study, we used a corrected *p*-value to reduce the possibility of type I statistical errors (false-positive findings), which may result in a slight difference of associations observed in two studies. If we used the naïve *p*-value in this study, several genes would be associated with duration of shedding (data not shown), similar to the previous study (Matussek et al., 2017). Furthermore, the reference databases and cutoffs for determination of virulence and AMR genes vary among different studies; thus, the data from different studies should be compared and interpreted with caution.

To conclude, this study provides pathogenomic insight into clinical STEC isolates and unravels multiplex genetic factors associated with clinical outcomes. Further studies are needed to elucidate the functions of these genetic factors as well as their interactions with host in STEC disease progression. These knowledge could contribute to monitoring STEC infections and management of severe clinical outcomes.

## Data Availability Statement

The datasets presented in this study can be found in online repositories. The names of the repository/repositories and accession number(s) can be found in the article/Supplementary Material.

## Ethics Statement

Ethical review and approval was not required for the study on human participants in accordance with the local legislation and institutional requirements. Written informed consent from the participants’ legal guardian/next of kin was not required to participate in this study in accordance with the national legislation and the institutional requirements.

## Author Contributions

AM and XB designed the study. XB, JZ, and YH analyzed the data and drafted the manuscript. CJ, IH, and SM collected the clinical and epidemiological data. NF, AA, and SM contributed to the statistics analysis. SL, NF, and YX provided their expertise feedback. All the authors reviewed the draft and contributed significantly to the final manuscript.

## Conflict of Interest

The authors declare that the research was conducted in the absence of any commercial or financial relationships that could be construed as a potential conflict of interest.

## Footnotes

1 https://github.com/s-andrews/FastQC

2 https://github.com/tseemann/abricate

3 http://www.mgc.ac.cn/VFs/

4 https://card.mcmaster.ca/

5 https://github.com/sanger-pathogens/Roary

6 https://github.com/jizhang-nz/fast-GeP

7 http://itol.embl.de
