# Supplementary material for: Genomic Insights Into Clinical Shiga Toxin-Producing Escherichia coli Strains: A 15-Year Period Survey in Jönköping, Sweden
Source: Front Microbiol. 2021 Feb 5;12:627861. doi: 10.3389/fmicb.2021.627861 (PMC7893091; doi:10.3389/fmicb.2021.627861)
Supplement: Supplementary file 1 [file Data_Sheet_1.PDF]

Supplementary Material

Table S1. Metadata of 184 clinical STEC isolates (.xlsx)

Table S2. Association between virulence genes and clinical symptoms (.xlsx)

Table S3. Presence of AMR genes in 184 STEC isolates (.xlsx)

Table S4. Accessory genes significantly associated with clinical symptoms (.xlsx)

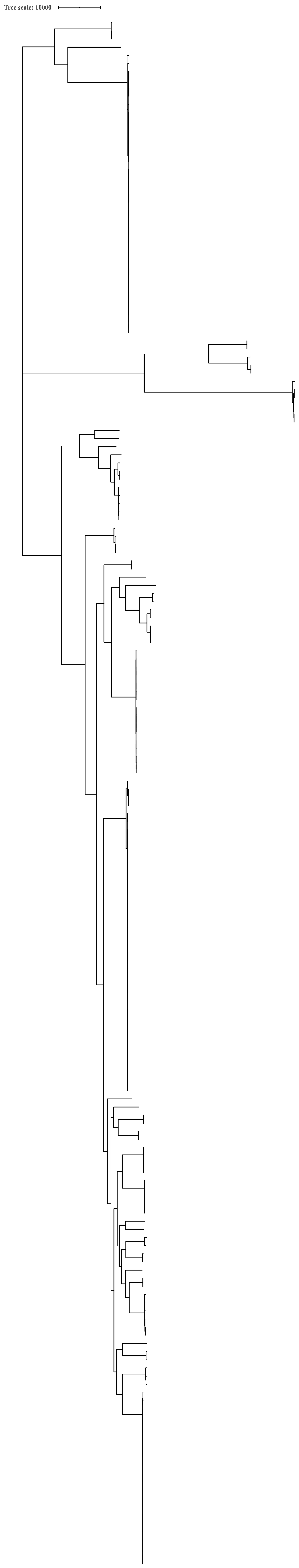

Isolate name	Serotype	*stx* subtype	*eae*	BD	NBS	HUS	Duration of shedding	Outbreak group
SE1406	O145:H28	stx2a	+	-	+	-	NA	NA
RM13514	O145:H28							
SE0714	O145:H28	stx1a	+	-	+	-	Short	NA
SE1311	O8:H19	stx1d	-	-	+	-	Short	55
SE1119	O157:H7	stx2c	+	-	+	-	Short	NA
SE1704	O157:H7	stx2c	+	-	+	-	Short	NA
SE0604	O157:H7	stx1a, stx2c	+	+	-	-	Short	NA
SE0909	O157:H7	stx2c	+	-	+	-	Long	NA
SE0809	O157:H7	stx2c	+	-	+	-	Short	29
SE0808	O157:H7	stx2c	+	-	+	-	Short	29
SE0803	O157:H7	stx2c	+	+	-	-	Short	NA
SE0503	O157:H7	stx1a, stx2c	+	+	-	-	Short	14
SE1208	O157:H7	stx1a, stx2c	+	+	-	-	NA	NA
SE0907	O157:H7	stx1a, stx2c	+	+	-	-	Short	NA
SE1204	O157:H7	stx1a, stx2c	+	+	-	-	NA	NA
SE0708	O157:H7	stx1a, stx2c	+	+	-	-	Long	NA
SE1302	O157:H7	stx1a, stx2c	+	-	+	-	Short	56
SE1303	O157:H7	stx1a, stx2c	+	-	+	-	Long	56
EDL933	O157:H7							
SE0304	O157:H7	stx1a, stx2a	+	+	-	-	NA	NA
SE0404	O157:H7	stx2c	+	-	+	-	Long	8
SE1308	O157:H7	stx2a, stx2c	+	-	+	-	NA	59
SE1309	O157:H7	stx2a, stx2c	+	-	+	-	NA	59
SE0715	O157:H7	stx2a, stx2c	+	+	-	-	NA	NA
SE0515	O157:H7	stx2a, stx2c	+	-	-	+	Long	19
SE0506	O157:H7	stx2a, stx2c	+	+	-	-	Short	NA
SE0303	O157:H7	stx2a, stx2c	+	+	-	-	Long	NA
SE0407	O157:H7	stx2a, stx2c	+	+	-	-	Long	NA
SE0412	O157:H7	stx2a, stx2c	+	+	-	-	NA	NA
SE0903	O157:H7	stx2a, stx2c	+	+	-	-	Long	35
SE1201	O157:H7	stx2a, stx2c	+	+	-	-	NA	NA
SE0311	O157:H7	stx2a, stx2c	+	-	+	-	Short	2
SE0309	O157:H7	stx2a, stx2c	+	-	+	-	Short	2
SE0310	O157:H7	stx2a, stx2c	+	-	+	-	Short	2
SE1315	O157:H7	stx2a, stx2c	+	+	-	-	NA	NA
SE0512	O157:H7	stx2a, stx2c	+	+	-	-	Short	NA
SE0510	O157:H7	stx2a, stx2c	+	+	-	-	Short	NA
SE0514	O157:H7	stx2a, stx2c	+	-	+	-	Short	16
SE0513	O157:H7	stx2a, stx2c	+	+	-	-	Short	NA
SE1502	O183:H18	stx1a, stx2a	-	+	-	-	NA	NA
SE1115	O183:H18	stx1a, stx2a	-	+	-	-	Long	NA
SE0401	O185:H28	stx1a, stx2a	+	+	-	-	Short	NA
SE1114	O146:H28	stx2b	-	-	+	-	Short	NA
SE1105	O146:H28	stx2b	-	-	+	-	NA	NA
SE0815	O156:H7	stx1a	-	-	+	-	NA	30
SE1211	O117:H7	stx1a	-	-	+	-	Long	53
SE1206	O117:H7	stx1a	-	-	+	-	Long	53
SE0409	O117:H7	stx1a	-	-	+	-	NA	NA
SE1412	O117:H7	stx1a	-	-	+	-	Long	NA
SE0707	O117:H7	stx1a	-	-	+	-	NA	NA
SE574	O136:H12	stx2a	-	-	+	-	Short	64
SE573	O15:H16	stx2g	-	-	+	-	Short	NA
SE0812	O35:H10	stx2a	-	+	-	-	Short	NA
SE1107	O180:H2	stx2a	+	-	+	-	NA	NA
SE575	O100:H30	stx2e	-	-	+	-	NA	NA
SE1307	O96:H19	stx1a	-	-	+	-	Long	NA
SE1306	O96:H19	stx1a	-	-	+	-	Long	NA
SE1003	O115:H10	stx1a	-	-	+	-	Short	NA
SE1106	O4:H16	stx1c	-	-	+	-	NA	NA
SE0905	O113:H4	stx2d	-	-	+	-	NA	NA
SE0805	O113:H4	stx1c, stx2b	-	-	+	-	Short	NA
SE0701	O113:H4	stx2b	-	-	+	-	Long	NA
SE0704	O8:H9	stx2c	-	-	+	-	Short	NA
SE0703	Ont:H4	stx1c	-	-	+	-	NA	NA
SE0605	O78:H4	stx1c, stx2b	-	-	+	-	NA	25
SE0606	O78:H4	stx1c, stx2b	-	-	+	-	Long	25
SE0902	O84:H2	stx1a	+	-	+	-	Long	NA
SE0408	O98:H21	stx1a	+	-	-	+	Short	NA
SE0706	O9:H21	stx2e	-	-	+	-	NA	NA
SE1602	O103:H2	stx1a	+	-	+	-	Short	NA
SE1404	O165:H25	stx2a, stx2c	+	-	+	-	Long	NA
SE1205	O165:H25	stx2a, stx2c	+	+	-	-	NA	NA
SE1604	O5:H9	stx1a	+	-	+	-	Short	NA
SE1706	O5:H9	stx1a	+	-	+	-	Long	74
SE0516	O177:H25	stx2c	+	-	+	-	Short	21
SE0518	O177:H25	stx2c	+	-	+	-	Long	12
SE0517	O177:H25	stx2c	+	-	+	-	Short	12
SE1001	O121:H19	stx2a	+	-	+	-	Short	NA
SE0501	O121:H19	stx1a, stx2a	+	-	+	-	Short	13
SE1705	O121:H19	stx2a	+	-	+	-	Long	NA
SE1108	O121:H19	stx2a	+	-	+	-	NA	NA
SE1606	O121:H19	stx2a	+	+	-	-	Long	NA
SE1503	O121:H19	stx2a	+	-	-	+	NA	67
SE1707	O121:H19	stx2a	+	+	-	-	NA	76
SE1005	O121:H19	stx2a	+	+	-	-	Short	NA
SE1401	O121:H19	stx2a	+	+	-	-	Long	61
SE1402	O121:H19	stx2a	+	+	-	-	Long	61
SE1403	O121:H19	stx2a	+	-	+	-	NA	61
SE0711	O121:H19	stx2a	+	+	-	-	Short	NA
SE0814	O121:H19	stx2a	+	+	-	-	NA	NA
SE0713	O121:H19	stx2a	+	-	+	-	Long	23
SE0712	O121:H19	stx2a	+	-	+	-	Long	23
SE0603	O121:H19	stx2a	+	+	-	-	Short	23
SE0901	O111:H8	stx1a	+	-	+	-	Short	NA
11128	O111:H-							
SE1316	O111:H8	stx1a	+	-	+	-	Short	60
SE1313	O111:H8	stx1a	+	+	-	-	Short	NA
SE1008	O26:H11	stx1a	+	-	+	-	Short	NA
SE1007	O26:H11	stx2a	+	-	+	-	Long	43
SE0504	O26:H11	stx1a	+	-	+	-	Long	NA
SE0601	O26:H11	stx1a	+	-	+	-	Long	NA
SE0505	O26:H11	stx1a	+	-	+	-	NA	15
SE0705	O26:H11	stx1a	+	-	+	-	Short	NA
SE1002	O26:H11	stx1a	+	-	+	-	Short	40
SE0511	O26:H11	stx1a	+	-	+	-	Short	17
SE0716	O26:H11	stx1a	+	-	+	-	Long	NA
SE0702	O26:H11	stx1a	+	+	-	-	Long	NA
11368	O26:H11							
SE1701	O26:H11	stx1a	+	+	-	-	Short	NA
SE0508	O26:H11	stx1a	+	-	+	-	Long	NA
SE1408	O26:H11	stx1a	+	+	-	-	Long	NA
SE1113	O26:H11	stx1a	+	-	+	-	Short	NA
SE0519	O26:H11	stx1a	+	-	+	-	NA	NA
SE1110	O26:H11	stx1a	+	-	+	-	NA	47
SE1116	O26:H11	stx2a	+	-	+	-	Long	NA
SE1213	O26:H11	stx2a	+	+	-	-	NA	NA
SE1111	O26:H11	stx1a	+	-	+	-	Short	NA
SE0806	O26:H11	stx1a	+	-	+	-	Long	32
SE0810	O26:H11	stx1a	+	-	+	-	Short	NA
SE1314	O26:H11	stx1a	+	-	+	-	Long	NA
SE1203	O26:H11	stx1a	+	+	-	-	Short	NA
SE1409	O26:H11	stx1a	+	+	-	-	NA	NA
SE1702	O26:H11	stx1a, stx2a	+	+	-	-	NA	NA
SE1209	O26:H11	stx1a	+	+	-	-	NA	NA
SE1608	O26:H11	stx1a	+	-	+	-	Short	NA
SE1312	O26:H11	stx1a	+	+	-	-	NA	NA
SE1405	O26:H11	stx1a	+	+	-	-	Short	NA
SE1504	O26:H11	stx1a	+	-	+	-	Long	66
SE1411	O26:H11	stx1a	+	-	+	-	Long	65
SE1410	O26:H11	stx1a	+	-	+	-	Long	65
SE1601	O26:H11	stx1a	+	+	-	-	NA	NA
SE1703	O26:H11	stx1a	+	-	+	-	NA	NA
SE0402	O5:H19	stx1c	-	-	+	-	NA	7
SE0709	O167:H14	stx1c	-	-	+	-	NA	NA
SE1210	O117:H8	stx1c	-	-	+	-	NA	53
SE0710	O117:H8	stx1c	-	-	+	-	NA	28
SE1301	O163:H19	stx1a, stx2d	-	-	+	-	Long	55
SE1310	O163:H19	stx1a, stx2d	-	-	+	-	Short	55
SE1214	O150:H10	stx2b	-	-	+	-	NA	50
SE1212	O150:H10	stx2b	-	-	+	-	Short	50
SE1215	O150:H10	stx2b	-	-	+	-	NA	50
SE1216	O150:H10	stx2b	-	-	+	-	NA	50
SE1103	O104:H4	stx2a	-	-	-	+	Long	NA
SE1101	O104:H4	stx2a	-	+	-	-	Short	NA
SE1104	O104:H4	stx2a	-	-	+	-	NA	NA
SE1102	O104:H4	stx2a	-	-	-	+	NA	NA
2011C-3493	O104:H4							
SE0811	O112ab:H2	stx2b, stx2d	-	-	+	-	Long	NA
SE0306	O15:H27	stx1c, stx2b	-	-	+	-	NA	5
SE0804	O175:H21	stx1a, stx2a	-	-	+	-	Long	NA
SE1117	O126:H20	stx2a	-	-	+	-	NA	NA
SE572	O187:H28	stx2g	-	-	+	-	Short	NA
SE1605	Ont:H21	stx1c	-	-	+	-	Long	NA
SE0602	O76:H19	stx1c, stx2d	-	-	+	-	Short	22
SE0406	O55:H12	stx1a	-	-	+	-	Long	9
SE0403	O55:H12	stx1a	-	-	+	-	Long	9
SE1207	O146:H21	stx1c, stx2b	-	-	+	-	NA	NA
SE0904	O146:H21	stx2b	-	+	-	-	Long	NA
SE0906	O146:H21	stx1c, stx2b	-	-	+	-	Long	NA
SE0301	O91:H21	stx2d	-	-	+	-	Short	NA
SE0410	O91:H21	stx2d	-	-	+	-	Short	6
SE0411	O91:H21	stx2d	-	-	+	-	Short	6
SE1709	Ont:H16	stx1c	-	-	+	-	Short	NA
SE0813	O91:H14	stx1a, stx2b	-	-	+	-	Long	33
SE0308	O91:H14	stx1a, stx2b	-	-	+	-	NA	NA
SE0307	O128ab:H2	stx1c, stx2b	-	-	+	-	NA	5
SE0302	O128ab:H2	stx1c	-	-	+	-	Long	4
SE0405	O128ab:H2	stx1c, stx2b	-	+	-	-	Long	10
SE1112	O123:H2	stx1a	+	-	+	-	Long	46
SE1109	O123:H2	stx1a	+	-	+	-	Long	44
SE1317	O123:H2	stx1a	+	-	+	-	Long	NA
12009	O103:H2							
SE1318	O71:H2	stx1a	+	-	+	-	Short	NA
SE0502	O103:H2	stx1a	+	-	+	-	Long	NA
SE1004	O103:H2	stx1a	+	-	+	-	Long	41
SE1006	O103:H2	stx1a	+	-	+	-	Short	41
SE1603	O103:H2	stx1a	+	-	+	-	Short	71
SE1305	O103:H2	stx1a	+	-	+	-	Short	57
SE1304	O103:H2	stx1a	+	+	-	-	Long	NA
SE1501	O103:H2	stx1a	+	-	+	-	NA	NA
SE1202	O103:H2	stx1a	+	-	+	-	Short	51
SE0802	O103:H2	stx1a	+	-	+	-	Short	31
SE0801	O103:H2	stx1a	+	-	+	-	Long	31
SE0509	O103:H2	stx1a	+	-	+	-	Long	16
SE0305	O103:H2	stx1a	+	+	-	-	Short	NA
SE0807	O103:H2	stx1a	+	+	-	-	Long	NA
SE1505	O103:H2	stx1a	+	-	+	-	Long	NA
SE1413	O103:H2	stx1a	+	+	-	-	Long	NA
SE1607	O103:H2	stx1a	+	-	+	-	NA	NA
SE1118	O103:H2	stx1a	+	-	+	-	Short	NA

Figure S1. Phylogenetic relatedness of 184 STEC isolates and reference strains

Gubbins tree generated with the concatenated sequences of all shared loci found in wgMLST analysis. Six reference STEC strains representative of predominant STEC serotypes are indicated in blue.

NA: not available.
